# The Lisbon patient: exceptional longevity with HIV suggests healthy aging as an ultimate goal for HIV care

**DOI:** 10.1186/s12879-020-05020-8

**Published:** 2020-04-20

**Authors:** Ines Pintassilgo, Matteo Cesari, Henrique N. Santos, Jovana Milic, Iacopo Franconi, Cristina Mussini, Nuno Marques, Giovanni Guaraldi

**Affiliations:** 1 Internal Medicine Department, Hospital Garcia de Orta, Almada, Portugal; 2 Department of Clinical and Community Sciences, Università di Milano, Milan, Italy; 3 Geriatric Unit, Fondazione IRCCS Ca’ Granda - Ospedale Maggiore Policlinico, Milan, Italy; 4 Infectious Diseases Department, Hospital Garcia de Orta, Almada, Portugal; 5 Modena HIV Metabolic Clinic, Azienda Policlinico-Universitaria di Modena, Modena, Italy; 6 Department of Medical and Surgical Sciences for Children & Adults, University of Modena and Reggio Emilia, Modena, Italy; 7 Clinical and Experimental Medicine PhD Program, University of Modena and Reggio Emilia, Modena, Italy

**Keywords:** Healthy aging, HIV, Intrinsic capacity, UNAID

## Abstract

In the context of global aging, HIV infection has become a new chronic disease and requires innovative models of care. Treating isolated comorbidities represents a useless and potentially harmful practice at advanced age. Therefore, a patient-centered approach, in which the interventions are focused on the biology and function of the individual, with understanding of the importance of securing social and home environment that provides psychosocial support, better suits unmet health needs. We present a paradigmatic case of healthy aging: the first reported HIV-infected patient who achieved 100th of life – the Lisbon patient. The construct of healthy aging, recently introduced by the World Health Organization, is the best example of this comprehensive model and could represent the fourth target of UNAIDS agenda of the end of AIDS.

## Background

Epidemiological models suggest that life expectancy of people living with HIV achieving immunological success is similar to that of the general population [1], nevertheless the clinical experience of extreme longevity is still limited.

This review gets inspiration from the first reported, and presumably not unique, 100-year-old HIV-infected person, to discuss new principles of geriatric medicine that could be applied in HIV setting. We will use this case as a paradigm to identify opportunities in joining HIV and geriatric medicine to improve the care of older people living with HIV (OPLWH). We obtained consent to present this case from patient’s daughter.

The hereby called “Lisbon patient” was diagnosed with HIV infection at the age of 84, and peacefully died in his sleep 4 months after turning 100 years.

The date of HIV acquisition was not known; at the time of diagnosis, he presented with non-Hodgkin lymphoma and CMV colitis. Nadir CD4 T cell count was < 100 c/μL.

He started antiretroviral therapy (ART) immediately after being diagnosed and was exposed to chemotherapy and toxic ART drugs, including zidovudine, stavudine and first-generation protease inhibitors. He has achieved an undetectable level of HIV-RNA since the beginning of HIV treatment up to death. His last available CD4+ T-lymphocyte count were 560 cells/μL (34%) with CD4/CD8 = 0.97.

From a geriatric perspective, the Lisbon patient had multi-morbidity with hypertension, liver steatosis, osteoarthrosis, and benign prostate hypertrophy. With regards to geriatric syndromes he had sarcopenia and was phenotypically frail [2], due to muscle weakness, slow gait speed, and sedentariness. He had no polypharmacy: the only drug he was taking (apart from ART) was antihypertensive medication. His cognitive function was normal (as estimated by MOCA score).

He had a fortunate genetic inheritance given that his father and siblings reached more than 90 years of age. He had been living in a good environment with no socio-economic difficulties with support and love of his daughter who took care of him (even though she is 75 years old).

## Main text

The number of OPLWH is increasing thanks to the synergistic result of two phenomena: people living with HIV live longer and more people acquire HIV at an older age [3]. The former is represented by OPLWH that have been longer exposed to antiretroviral regimens with harmful metabolic effects leading to accentuated risk for co-morbidities, while the latter comprises OPLWH with lower perception of sexual risk that might have developed co-morbidities that are not HIV-associated [4, 5].

To better characterize the **diversity** of OPLWH, aging cohorts are rising across Europe to address similarities and differences with the **complexity** of aging trajectories in the general population [5].

In the context of global aging, HIV infection represents a new chronic disease in which the principles of geriatric medicine should be applied. Relevant clinical outcomes go far beyond immune-virologic parameters or even age-related non-infectious co-morbidities alone [6] and include geriatric syndromes. They are multifactorial health conditions that occur when the accumulated deficits in multiple systems, at a clinical level most commonly represented by frailty. It describes a lack of homeostatic reserves exposing the individual to a higher risk of negative outcomes [7].

Frailty assessment allows to identify the causes of individual’s increased vulnerability and implement a person-tailored intervention plan, called comprehensive geriatric assessment.

The new EACS guidelines recommend screening of OPLWH for frailty in the context of a Comprehensive Geriatric Assessment (CGA) [8], defined as a multidisciplinary diagnostic and treatment process that identifies medical, psychosocial, and functional limitations of a frail older person to develop a coordinated plan to maximize overall health with aging [6, 9].

Healthy aging was described in the World report on Aging and Health, developed by World Health Organization, as the process of developing and maintaining the functional ability that enables well-being in older age [10].. This construct relies on the interplay between intrinsic capacity (IC), that includes all physical and cognitive functions of an individual, and the environment. Healthy aging is influenced by our genetic inheritance and personal characteristics such as sex, ethnicity, education and occupation [10]. Assessing IC is both a multidisciplinary and multidimensional process, designed to evaluate the individual’s characteristics on the basis of five functional domains: locomotion, cognitive, psychologic, vitality-related and sensory [11].

Moreover, the IC might be considered as an evolution of the frailty concept, as it takes into special consideration functional reserves expressed by vitality domain, the need for a worldwide implementation of prevention, the continuum of the aging process [11]. This novel approach provides a few advantages over frailty as it gives a positive connotation to the aging phenomenon, provides a possibility to better describe aging trajectories instead of using cross-sectional cut-points and promotes self-empowerment of the individual for his/her health [12].

Both frailty and IC are based on the assumption that the aging individual need to be assessed and managed in a patient centered model through integration of multidisciplinary health services and data obtained by the health environment of the subject [12].

This geriatric approach changes the medical perspective from disease management to preservation of health status maintaining the individual’s functional ability, thus having the capabilities that enable all people to be and do what they have reason to value. This includes a person’s ability to meet their basic needs, to learn, grow and make decisions, to be mobile, to build and maintain relationships, and to contribute to society [10].

IC domains, except for sensory function, were all preserved in the Lisbon patient. Considering the environmental part of healthy aging, we can say that the Lisbon patient had been living in a favorable environment that allowed him to achieve longevity. He taught us that it is possible experience healthy aging regardless of being affected by HIV as a chronic condition. We might indeed consider our ‘Lisbon patient’ as an icon inspiring scientific and community commitment.

However, there is still a lot to be done, as we are seeking for the reliable healthy aging and IC tool. The WHO has launched the Integrated Care for Older People (ICOPE) guidelines with the aim to assist health care professionals to identify declines in physical and mental capacity and to deliver effective interventions to prevent and delay progression of disease and disability [13, 14]. Health care services should meet the needs of those with declining IC, but also of those with preserved IC.

In HIV scenario, OPLWH may present a similar viro-immunological status, but completely different health needs that require different intensity of care [14]. This approach leads to tailored interventions taking into an account management of ART and co-morbidities, social care and preservation of IC [15].

The Joint United Nations Program on HIV/AIDS (UNAIDS) launched the agenda for the end of AIDS introducing the “90–90–90 targets” [16]. This program introduces an equity principle for HIV care. Lazarus et al. suggested to add a fourth 90% in the 90–90-90 cascade paradigm: 90% of all virally suppressed patients should reach good quality of life [17]. The idea of introducing quality of life as the major outcome in people living with HIV originates from the era of first ART regimens, when it was proposed as a part of the evaluation of new and existing treatment strategies [18].

## Conclusions

In a patient related outcome context, quality of life may represent a valuable contribution focusing on the subjective perception of the individual’s status and implicitly based on his socio-cultural and economic environment. The limit of this patient-related outcome is that it may fail to represent a robust and standardized parameter for driving public health decisions. Furthermore, quality of life is not necessarily related to the functional competence of the aging individual, as depicted by the Lisbon Patient’s suboptimal quality of life, assessed by the EQ-5D-5 L questionnaire [19].

On the other hand, healthy aging, although designed for the general population, can perfectly fit to OPLWH, as it reinforces a patient-centered approach and asks for a reshaped healthcare model. An integrated model that includes community, the patient, and healthcare providers could identify both medical and social needs, and increase the possibilities for effective intervention [14].

The Lisbon patient commits us to be ambitious to add healthy aging as the fourth target to the UNAIDS 90–90-90 program.

## Data Availability

All data generated or analyzed during this study are included in this published article.

## Abbreviations

AIDS: Acquired immune deficiency syndrome
ART: Antiretroviral therapy
CMV: Cytomegalovirus
HIV: Human immunodeficiency virus
ICOPE: Integrated Care for Older People
MOCA: The Montreal Cognitive Assessment
OPLWH: Older people living with HIV
UNAIDS: United Nations Program on HIV/AIDS
WHO: World Health Organization

## References

[1] Smit M, Brinkman K, Geerlings S (2015). Future challenges for clinical care of an ageing population infected with HIV: a modelling study. Lancet Infect Dis United States.

[2] Fried LP, Seeman T, Newman AB (2001). Frailty in older adults: evidence for a phenotype. J Gerontol Ser A Biol Sci Med Sci.

[3] Chambers LA, Wilson MG, Rueda S, Gogolishvili D, Shi MQ, Rourke SB (2014). Evidence informing the intersection of HIV, aging and health: a scoping review. AIDS Behav United States.

[4] Cooperman NA, Arnsten JH, Klein RS (2007). Current sexual activity and risky sexual behavior in older men with or at risk for HIV infection. AIDS Educ Prev United States.

[5] Milic J, Russwurm M, Cerezales Calvino A, Brañas F, Sánchez-Conde M, Guaraldi G (2019). European cohorts of older HIV adults: POPPY, AGEhIV, GEPPO, COBRA and FUNCFRAIL. Eur Geriatr Med.

[6] Koroukian SM, Schiltz N, Warner DF (2016). Combinations of chronic conditions, functional limitations, and geriatric syndromes that predict health outcomes. J Gen Intern Med United States.

[7] Cesari M, Calvani R, Marzetti E (2017). Frailty in older persons. Clin Geriatr Med United States.

[8] 10th EACS Guidelines. Available at: https://www.eacsociety.org/files/2019_guidelines-10.0_final.pdf (Accessed 24th December 2019).

[9] Erlandson KM, Schrack JA, Jankowski CM, Brown TT, Campbell TB (2014). Functional impairment, disability, and frailty in adults aging with HIV-infection. Curr HIV/AIDS Rep United States.

[10] World report on ageing and health 2015. World rep ageing heal 2015. Available at https://wwwwhoint/ageing/events/world-report-2015-launch/en/ Last access: 24 Dec 2019.

[11] Cesari M, Araujo de Carvalho I, Amuthavalli Thiyagarajan J (2018). Evidence for the domains supporting the construct of intrinsic capacity. J Gerontol A Biol Sci Med Sci United States.

[12] Belloni G, Cesari M (2019). Frailty and intrinsic capacity: two distinct but related constructs. Front Med.

[13] de Carvalho IA, Epping-Jordan J, Beard JR (2019). Integrated Care for Older People.

[14] Guaraldi G, Milic J, Wu AW (2019). What is the measure of success in HIV? The fourth 90: quality of life or healthy aging?. Eur Geriatr Med Springer International Publishing.

[15] Guaraldi G, Milic J (2019). The interplay between frailty and intrinsic capacity in aging and HIV infection. AIDS Res Hum Retroviruses United States.

[16] Sidibe M (2018). UNAIDS Data 2018. Program HIV/AIDS.

[17] Lazarus JV, Safreed-Harmon K, Barton SE (2016). Beyond viral suppression of HIV - the new quality of life frontier. BMC Med England.

[18] Wu AW (2000). Quality of life assessment comes of age in the era of highly active antiretroviral therapy. Aids.

[19] Devlin NJ, Brooks R (2017). EQ-5D and the EuroQol Group: Past, Present and Future. Appl Health Econ Health Policy. New Zealand.

